# RAC1 GTPase plays an important role in γ-irradiation induced G_2_/M checkpoint activation

**DOI:** 10.1186/bcr3164

**Published:** 2012-04-11

**Authors:** Ying Yan, Patrick M Greer, Phu T Cao, Ryan H Kolb, Kenneth H Cowan

**Affiliations:** 1 Eppley Institute for Research in Cancer and Allied Diseases, University of Nebraska Medical Center, 986805 Nebraska Medical Center, Omaha, NE, USA

## Abstract

**Introduction:**

In response to gamma-irradiation (IR)-induced double-strand DNA breaks, cells undergo cell-cycle arrest, allowing time for DNA repair before reentering the cell cycle. G_2_/M checkpoint activation involves activation of ataxia telangiectasia mutated (ATM)/ATM- and rad3-related (ATR) kinases and inhibition of Cdc25 phosphatases, resulting in inhibition of Cdc2 kinase and subsequent G_2_/M cell-cycle arrest. Previous studies from our laboratory showed that the G_2_/M checkpoint activation after IR exposure of MCF-7 breast cancer cells is dependent on the activation of extracellular signal-regulated protein kinase 1 and 2 (ERK1/2) signaling. In the present studies, we investigated the role of Ras-related C3 botulinum toxin substrate 1 (Rac1) guanosine triphosphatase (GTPase) in IR-induced G_2_/M checkpoint response and ERK1/2 activation, as well as in cell survival after IR.

**Methods:**

With Rac1-specific inhibitor, dominant negative mutant Rac1 (N17Rac1) and specific small interfering RNA, the effect of Rac1 on IR-induced G_2_/M checkpoint response and ERK1/2 activation was examined in human breast cancer cells. In addition, the effect of Rac1 on cell survival after irradiation was assessed by using Rac1-specific inhibitor.

**Results:**

IR exposure of MCF-7 breast cancer cells was associated with a marked activation of Rac1 GTPase. Furthermore, inhibition of Rac1 by using specific inhibitor, dominant-negative Rac1 mutant, or specific siRNA resulted in attenuation of IR-induced G_2_/M arrest and concomitant diminution of IR-induced activation of ATM, ATR, Chk1, and Chk2 kinases, as well as phosphorylation of Cdc2-Tyr15. Moreover, Rac1 inhibition or decreased Rac1 expression also abrogated IR-induced phosphorylation of mitogen-activated protein kinase kinase 1 and 2 (MEK1/2) and ERK1/2. Ultimately, inhibition of Rac1 markedly increased cellular sensitivity to IR exposure, which involves induction of apoptosis.

**Conclusion:**

Studies in this report suggest that Rac1 GTPase plays an essential role in the activation of IR-induced ERK1/2 signaling and subsequent G_2_/M checkpoint response. Furthermore, results also support a role for Rac1 in promoting cell survival after irradiation treatment.

## Introduction

DNA damage by ionizing irradiation (IR) triggers rapid activation of DNA-damage checkpoint response, resulting in either cell-cycle arrest that allows DNA repair or induction of apoptosis, which eliminates seriously damaged or deregulated cells [1]. Previous studies identified several intracellular signaling cascades, including signalings mediated by ataxia telangiectasia-mutated (ATM) and ATM- and rad3-related (ATR), in the activation of DNA-damage checkpoint response [2].

The G_2_/M cell-cycle checkpoint is tightly controlled by the Cdc2/cyclin B complex, whose activity is required for G_2_/M transition of the cell cycle [3]. Previous studies identified the Cdc2-Tyr15 as a critical site involved in G_2_/M-checkpoint control in response to DNA damage. Cdc2-Tyr15 phosphorylation is induced and maintained during radiation-induced G_2_/M arrest, and introduction in fission yeast of a mutant Cdc2-Y15F, which cannot be phosphorylated at the tyrosine 15 residue, completely abolished DNA-damage-induced G_2_/M arrest [4-6]. Cdc2-Tyr15 is phosphorylated by Wee1 kinase, which phosphorylates Cdc2 at Tyr15, and by Myt1 kinase, which phosphorylates Cdc2 at Thr14 and, to a lesser extent, at Tyr15 [7,8].

Dephosphorylation of Cdc2-Tyr15 involves Cdc25 dual-specific phosphatases [9]. In response to DNA damage, ATM and ATR kinases are rapidly activated through phosphorylation, which, in turn, leads to the phosphorylation/activation of their downstream targets Chk1 and Chk2 kinases, respectively. Activation of Chk1 and Chk2 kinases results in phosphorylation of Cdc25, leading to the subcellular sequestration, degradation, and/or inhibition of the Cdc25 phosphatases that normally activate Cdc2/cyclin B at the G_2_/M boundary [10].

On cell transition from G_2 _to mitotic phase, histone H3 is phosphorylated at Ser10, which is associated with chromosome condensation before cell division [11]. Because both G_2 _and mitotic cells have *4N*-DNA content and are not distinguishable from each other by propidium iodide staining, phosphorylation of H3-Ser10 in *4N*-DNA content cells has been commonly used as a specific marker indicative of mitotic cells [12]. Furthermore, previous studies indicate that the initial phosphorylation of H3-Ser10 occurs in the late G_2 _phase but only on the pericentromeric chromatin. As cells progress through mitosis, the phosphorylation spreads along chromosomes and is completed at the end of prophase [13,14]. Thus, a gradual increase in H3-Ser10 phosphorylation occurs from the beginning of mitosis to the end of mitosis. In log-phase growing cells, phosphorylation of H3-Ser10 in mitotic cells is detected in a wide range with flow-cytometry analysis [15,16]. In response to irradiation-induced G_2_/M cell-cycle arrest, the phosphorylation of H3-Ser10 is suppressed in irradiated cells because of the blockage of the G_2_/M transition of the cell cycle [3,15,16].

Previous studies in a wide variety of cell types have shown that IR exposure results in rapid activation of MAPK family members, including ERK1/2, JNK, and p38 [17,18]. Although p38γ activation may be essential in IR-induced G_2_/M arrest in HeLa and U2OS cells [19], studies from our laboratory and others have demonstrated that IR-induced ERK1/2 activation is necessary for the activation of the G_2_/M checkpoint response in MCF-7 breast cancer cells and that inhibition of ERK1/2 is associated with increased sensitivity to DNA-damaging agents [16,20,21].

Ras-related C3 botulinum toxin substrate 1 (Rac1), a member of the Rho family of small guanosine triphosphatases (GTPases), has been shown to play a critical role in the regulation of cytoskeleton reorganization, cell migration, and cell survival [22]. Rac1 overexpression has been detected in many tumor types, including breast, lung, and colon cancer [23-25]; and Rac1b, a fast-cycling splice variant of Rac1, has been observed to be highly expressed in some breast and colon cancers [25,26]. Through interaction with various downstream effectors, Rac1 has been shown to activate numerous signaling pathways, including those mediated by the members of the MAPK family [27]. In response to various stimuli, previous studies showed that Rac1 can activate ERK1/2 signaling via p21-activated kinases 1 and 2, which phosphorylate Raf1 and MEK1 and facilitate the formation of the Raf/MEK/ERK complex [28-30]. Other studies indicated that Rac1 is involved in the activation of JNK and p38 signaling in response to angiotensin II stimulation [31]. Although the regulation of Rac1 on cytoskeleton reorganization and cell migration has been intensively investigated, the contribution of Rac1 to cell-cycle regulation has remained largely unknown. A previous study showed that expression of N17Rac1, a dominant-negative mutant of Rac1, in log-phase growing Rat 2 fibroblast cells, resulted in G_2_/M cell-cycle arrest [32]. Furthermore, a recent report detected the presence of Rac1 in the nucleus, and the level of nuclear Rac1 was increased when cells were in late G_2 _phase [33]. This evidence suggests a potential role for Rac1 in the regulation of cell-cycle progression in proliferating cells.

In the present study, we examined the effect of Rac1 on the IR-induced G_2_/M checkpoint response in human breast cancer cells. Results presented in this report indicate that IR exposure of cells induces Rac1 activation and that this is necessary for the activation of ERK1/2 signaling, subsequent G_2_/M checkpoint response, and cell survival after IR.

## Materials and methods

### Cell culture and treatment

Human breast cancer cell lines MCF-7, T47D, ZR-75-1, and MDA-MB-231 were obtained from American Type Culture Collection (Manassas, VA, USA). MCF-7, T47D, and ZR-75-1 cells were maintained in Dulbecco Modified Eagle medium containing 10% fetal bovine serum. MDA-MB-231 cells were maintained in the Leibovitz L-15 medium containing 10% fetal bovine serum. MCF-10A is a nontumorigenic human mammary epithelial cell line that was spontaneously immortalized previously [34]. 76 N is a nontransformed line of primary human mammary epithelial cells immortalized by human telomerase (hTERT) [35]. MCF-10A and 76 N cells are kind gifts from Dr. Vimla Band (University of Nebraska Medical Center). Both cell lines were maintained in Dana-Farber Cancer Institute 1 growth medium (DFCI-1). DFCI-1 medium consists of α-MEM/Ham nutrient mixture F-12 (1:1, vol/vol) supplemented with epidermal growth factor (12.5 ng/ml), triiodothyronine (10 n*M*), Hepes (10 m*M*), ascorbic acid (50 μ*M*), estradiol (2 n*M*), insulin (1 μg/ml), hydrocortisone (2.8 μ*M*), ethanolamine (0.1 m*M*), phosphoethanolamine (0.1 m*M*), transferrin (10 μg/ml), L-glutamine (2 m*M*), sodium selenite (15 n*M*), cholera toxin (1 ng/ml), 1% fetal bovine serum, and bovine pituitary extract (35 μg/ml) [35].

Rac1-specific inhibitor NSC23766 [36] was obtained from Tocris Biosciences (Ellisville, MO, USA) and dissolved in water. For experiments involving IR exposure, exponentially growing cells were treated with IR and then incubated at 37°C for the indicated time before analysis. For experiments involving treatment with both NSC23766 and IR, cells were incubated with NSC23766 for 1 hour before IR exposure.

### Antibodies and recombinant proteins

All antibodies were obtained from Santa Cruz Biotechnology (Santa Cruz, CA, USA), unless otherwise indicated. These included mouse IgG for ATM (2C1) (Novus Biologicals, Littleton, CO, USA), Cdc2 (17), Chk1 (G-4), Chk2 (B-4), PARP (Ab-2) (EMD Biosciences, San Jose, CA, USA), phospho-ERK1/2 (E-4); rabbit IgG for ATM (Ab-3) (EMD Biosciences), Cdc2 (C-19), Chk1 (FL-476), Chk2 (Cell Signaling, Danvers, MA, USA) MEK1/2 (12-B), Rac1 (C-14); and goat IgG for Actin (I-19), ATR (N-19), phospho-Cdc2 (Tyr15), ERK1/2 (C-14-G), and phospho-MEK1/2 (Ser118/Ser122).

Recombinant PAK-1 protein for Rac1 activity assay was obtained from Addgene (Cambridge, NH, USA) as a glutathione *S*-transferase (GST) fusion protein containing full-length human PAK1 protein. Recombinant p53 protein for ATM and ATR kinase assays was a glutathione *S*-transferase (GST) fusion protein containing full-length human p53 (Addgene, Cambridge, MA, USA). Recombinant Cdc25C protein, the substrate for Chk1 and Chk2 kinase assay, was a GST fusion protein containing residues 200 to 256 of human Cdc25C (kindly provided by Dr. Helen Piwnica-Worms, Washington University School of Medicine). All GST fusion proteins were purified as described previously [16]. GST was used as a control substrate in all kinase assays and was prepared according to standard procedures (GE Healthcare Bio-Sciences, Piscataway, NJ, USA).

### Immunoblotting, immunoprecipitation, and kinase assay

Immunoblotting, immunoprecipitation, and kinase assays were performed as described previously [16,37,38]. Specific protein signals on Western blots were visualized by chemiluminescence exposed to x-ray film, scanned by using EPSON Perfection 4490PHOTO scanner, and analyzed by using the ImageJ analytical program (NIH, Bethesda, MD, USA).

### Rac1 activity assay

Rac1 activity was assayed by using a Rac1 assay kit (Upstate Biotechnology, Lake Placid, NY, USA), as described previously [39,40]. In brief, cells were lysed at 4°C in 25 m*M *HEPES buffer (pH 7.4) containing 10 m*M *MgCl_2_, 150 m*M *NaCl, 1% NP-40, 1 m*M *EDTA, 2% glycerol, 1 m*M *DTT, 1 μg/ml aprotinin, 1 μg/ml leupeptin, 1 μg/ml pepstatin, 1 m*M *phenylmethylsulfonyl fluoride, 1 m*M *sodium fluoride, and 1 m*M *sodium vanadate. Cell lysates were incubated with GST-PAK1 fusion protein for 1 hour to capture GTP-bound Rac1. The obtained GTP-bound Rac1 (Rac1-GTP) was resolved on a 4% to 20% SDS-PAGE and assessed with immunoblotting by using an anti-Rac1-specific antibody, as described by the manufacturer's instructions. As a positive control, MCF-7 cells were serum-starved for 24 hours in the medium containing 0.3% fetal bovine serum, treated with 1 μ*M *phorbol 12-myristate 13-acetate (PMA) for 5 minutes, and analyzed for Rac1 activity [41].

### Cell-cycle analysis

Fluorescence-activated cell sorting (FACS) analysis was performed on 20,000 cells by using a FACS Calibur instrument (Beckon Dickinson, Mansfield, MA, USA), as described previously [16].

### Analysis for mitotic cells

MCF-7 cells were exposed to IR in the presence/absence Rac1-specific inhibitor NSC23766, harvested at the indicated times, fixed in 70% ethanol, and stained with propidium iodide (PI) and anti-phospho-histone H3 antibody (Upstate Biotechnology, Lake Placid, NY, USA) [12]. Mitotic cells, which contain both *4N*-DNA content and phospho-histone H3, were determined by using a FACSCalibur instrument (Beckon Dickinson) and analyzed by using CELLQUEST software. Each analysis was performed by using 20,000 cells.

### The siRNAs and transfection

Short interfering RNA (siRNA) duplexes were obtained from Dharmacon Research (Chicago, IL, USA). Control nontargeting siRNA contains at least four mismatches to any human, mouse, or rat gene, as previously determined by the manufacturer. The sequence for Control-siRNA is 5'-UAAG GCUA UGAA GAGA UAC-3'. SMARTpool siRNAs targeting Rac1 consist of four siRNAs targeting multiple sites on Rac1 (Rac1-siRNAs). The sequences for Rac1-siRNAs are 5'-UAAG GAGA UUGG UGCU GUA-3', 5'-UAAA GACA CGAU CGAG AAA-3', 5'-CGGC ACCA CUGU CCCA ACA-3', and 5'-AUGA AAGU GUCA CGGG UAA-3'.

Cells were transfected with siRNAs at 100 n*M *by using Dharma*FECT*1 siRNA transfection reagent (Dharmacon Research, Chicago, IL, USA), according to the manufacturer's instruction. For experiments involving both siRNA transfection and IR exposure, transfected cells were first incubated for the indicated times and then exposed to IR.

### Adenoviral vectors and adenoviral infections

Recombinant adenovirus N17Rac1 (Ad.N17Rac1) and control adenovirus dl312 (Ad.Control) were kindly provided by Dr. Toren Finkel (NIH, Bethesda, MD, USA). In Ad.N17Rac1, the Rac1 cDNA contains a Ser-to-Asp substitution at position 17 and functions as a dominant-negative mutant [42].

Log-phase MCF-7 cells were infected at 50 PFU/cell with either Ad.N17Rac1 or Ad.Control for 24 hours before exposure to IR, as described previously [43]. For studies involving cell-cycle analysis, the cells were incubated for additional 24 hours after IR and analyzed for DNA content with flow cytometry [16]. For studies involving mitotic cell analysis, the irradiated cells were incubated for 2 hours and analyzed for cells containing both *4N*-DNA content and histone H3-Ser10 phosphorylation [12].

### DAPI staining

Apoptosis was assessed with 4',6-diamidino-2-phenylindole (DAPI) staining, as described previously [43]. Apoptotic cells were identified by condensation and fragmentation of nuclei [44]. The percentage of viable cells was calculated as the ratio of live cells to total cells counted. At least 800 cells were counted per sample.

### Cell-survival assay

Cell-survival assays were performed as described previously [45]. In brief, log-phase growing cells were exposed to IR at the doses indicated, incubated for 7 days, and visualized for viable cells by staining with crystal violet (Sigma-Aldrich, St. Louis, MO, USA). For experiments involving treatment with both NSC23766 and IR, cells were preincubated for 1 hour with 100 μ*M *NSC23766, exposed to IR, and incubated for an additional 3 hours after IR. The cells were washed and incubated in regular growth medium for 7 days before analysis. The obtained sample dishes were scanned on an EPSON Perfection 4490PHOTO scanner, and the amount of cells remaining on the culture dish was quantified by using the ImageJ analytic program.

### Clonogenic assay

Clonogenic assay was performed as described previously [46]. In brief, in the presence or absence of 100 μ*M *NSC23766, MCF-7 cells were exposed to IR at the doses indicated and incubated for 3 hours after IR. The cells were then rinsed with DMEM, reseeded at the cell number indicated in duplicate, and incubated for 10 to 14 days until colonies formed. The colonies were visualized with crystal violet staining and quantified by using ImageJ software, as described previously [47].

## Results

### IR exposure induces G_2_/M arrest and Rac1 GTPase activation in MCF-7 breast cancer cells

**Figure 1:**
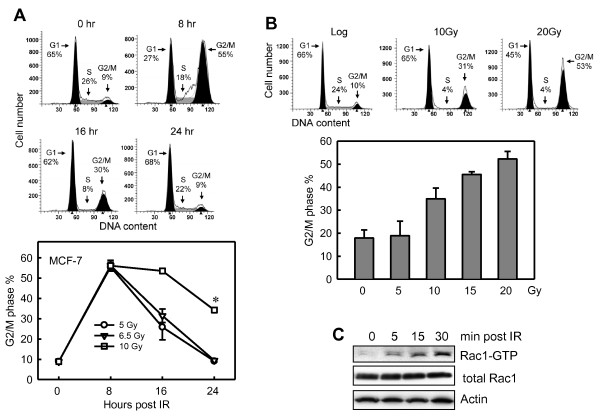
**IR induces G_2_/M cell-cycle arrest and Rac1 activation in MCF-7 cells**. **(A) **Upper panel: log-phase growing MCF-7 cells were exposed to 5-Gy IR, incubated for the indicated hours, and analyzed for cell cycle by fluorescence-activated cell sorting (FACS). Amounts of cells in the G_1_, S, and G_2_/M phases of the cell cycle are indicated. Lower panel: MCF-7 cells were exposed to IR at the dose indicated, incubated for the indicated hours, and analyzed for DNA content. Results depict the percentage of cells with *4N*-DNA content (G_2_/M phase) and represent the mean ± SD of two sets of experiment in duplicate samples. *P = < 0.001 (*n *= 4), significant difference from control unirradiated cells. **(B) **Upper panel: MCF-7 cells were exposed to IR at the indicated doses, incubated for 24 hours, and analyzed for cell cycle with FACS. Percentage of cells in G_1_, S, and G_2_/M phases of the cell cycle are indicated. Lower panel: Cells were exposed to increasing doses of IR, incubated for 24 hours, and analyzed for DNA content with FACS. Results depict the percentage of cells with 4*N*-DNA content and represent the mean ± SD of triplicate samples. **(C) **MCF-7 cells were exposed to 20-Gy IR, incubated for the indicated times, and analyzed for Rac1 activity, as described in Materials and methods.

To study the mechanisms regulating G_2_/M cell-cycle checkpoint response after IR exposure, log-phase growing MCF-7 cells were exposed to IR at the indicated doses and analyzed for DNA content at 8, 16, and 24 hours after IR. As shown in Figure 1A, IR exposure of MCF-7 cells resulted in marked increase in amount of *4N*-DNA content cells at 8 hours after IR, indicative of G_2_/M arrest [3]. Furthermore, the strength of the G_2_/M arrest detected at 8 hours after IR is independent of the IR dose used. At 24 hours after irradiation, the percentage of *4N*-DNA content cells in the samples treated with 5-Gy or 6.5-Gy was returned to baseline, whereas the percentage of *4N*-DNA content cells in the 10-Gy-treated samples remained significantly above baseline.

We next quantified the amount of *4N*-DNA content cells in MCF-7 cells exposed to increasing doses of IR and incubated for 24 hours. As shown in Figure 1B, at 24 hours after IR, the increase in amount of *4N*-DNA content cells in irradiated cells was dose dependent. Samples exposed to 20-Gy IR and incubated for 24 hours revealed a fivefold increase in the amount of *4N*-DNA content cells compared with unirradiated control cells (Figure 1B).

We next assessed the changes in Rac1 activity in cells exposed to irradiation. As shown in Figure 1C, Rac1 activity was increased within 5 minutes after IR exposure of MCF-7 cells. At 30 minutes after IR exposure, an 18-fold increase in Rac1 activity was found in irradiated cells compared with control nonirradiated cells. Furthermore, the increase in Rac1 activity was noted for at least 1 hour after exposure to IR (data not shown).

### Rac1 activation is required for IR-induced G_2_/M cell-cycle arrest

**Figure 2:**
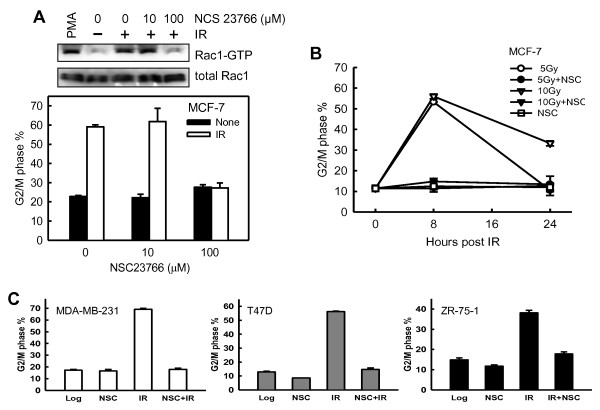
**Rac1 inhibition attenuates IR-induced G_2_/M arrest**. **(A) **Upper panel: MCF-7 cells were incubated for 1 hour with increasing doses of the Rac1 inhibitor NSC23766 and then exposed to 20-Gy IR. After 15-minute incubation at 37°C, Rac1 activity was determined. As a positive control, MCF-7 cells were serum-starved for 24 hours in the medium containing 0.3% fetal bovine serum, treated with 1 μ*M *phorbol 12-myristate 13-acetate for 5 minutes, and analyzed for Rac1 activity (PMA). Lower panel: MCF-7 cells were preincubated for 1 hour with NSC23766 at the indicated doses and treated with/without 20-Gy IR. After 24-hour incubation after IR, the cells were analyzed for DNA content with fluorescence-activated cell sorting (FACS). Graph depicts the percentage of cells with *4N*-DNA content and represents the mean ± SD of quadruplicate samples. **(B) **MCF-7 cells were exposed to IR at the indicated doses in the presence or absence of 100 μ*M *NSC23766. After IR, the cells were incubated for 8 or 24 hours and analyzed for DNA content. Results depict the percentage of cells with *4N*-DNA content and represent the mean ± SD of two separate experiments with duplicate samples. **(C) **Human breast cancer cells (MDA-MB-231, T47D, ZR-75-1) were treated with or without 10-Gy IR in the presence or absence of 100 μ*M *NSC23766, incubated for 24 hours, and analyzed for DNA content. Results depict the percentage of cells with *4N*-DNA content and represent the mean ± SD of two sets of experiments in duplicate samples.

By using a Rac1-specific inhibitor NSC23766 [36], we examined the effect of Rac1 on IR-induced G_2_/M arrest. For these experiments, MCF-7 cells were incubated for 1 hour in the presence of increasing doses of NSC23766 before exposure to 20-Gy IR. As shown in Figure 2A (upper panel), preincubation of MCF-7 cells with 100 μ*M *NSC23766 resulted in 90% inhibition in IR-induced Rac1 activity (*Rac1-GTP*). As shown in Figure 2A (lower panel), incubation of MCF-7 cells in the presence of 100 μ*M *NSC23766 resulted in a near-complete inhibition in IR-induced G_2_/M arrest (open bars). In contrast, incubation with NSC23766 alone in the absence of IR had only a subtle, if any, effect on the percentage of *4N*-DNA content cells relative to log-phase growing cells (Figure 2A, lower panel: solid bars). Furthermore, preincubation of cells in the presence of 10 μ*M *NSC23766, a dose that did not inhibit Rac1 activity, had no effect on IR-induced G_2_/M arrest (Figure 2A).

We next examined the effect of NSC23766 on the induction of G_2_/M arrest over time. For these studies, MCF-7 cells were exposed to 5-Gy or 10-Gy IR in the presence or absence of 100 μ*M *NSC23766, and the percentage of cells in G_2_/M phase was examined over time. As shown in Figure 2B, treatment of cells with 5-Gy and 10-Gy IR induced a marked increase in percentage of cells with G_2_/M DNA content at 8 hours after irradiation. However, this IR-induced G_2_/M arrest was completely attenuated by incubation of cells in the presence of Rac1 inhibitor NSC23766 (8 hours after IR, solid circle and triangle versus open circle and triangle). Furthermore, whereas 10-Gy-irradiated cells incubated in the absence of Rac1 inhibitor showed a threefold increase in percentage of G_2_/M DNA content cells at 24 hours after IR compared with control unirradiated cells, cells exposed to 10-Gy IR and incubated in the presence of NSC23766 showed no increase in the amount of G_2_/M DNA-content cells compared with the control cells (Figure 2B). Cells treated with NSC23766 alone in the absence of IR showed no increase in amount of G_2_/M DNA-content cells at all time points tested (open square). These results suggest a requirement for Rac1 activity in IR-induced G_2_/M cell-cycle arrest.

To confirm the results obtained from MCF-7 cells, we examined the effect of NSC23766 on IR-induced G_2_/M arrest in MDA-MB-231, T47D, and ZR-75-1 human breast cancer cells. As shown in Figure 2C, preincubating each of these cells with 100 μ*M *NSC23766 before exposure to 10-Gy IR resulted in a marked attenuation of the IR-induced G_2_/M cell-cycle arrest.

**Figure 3:**
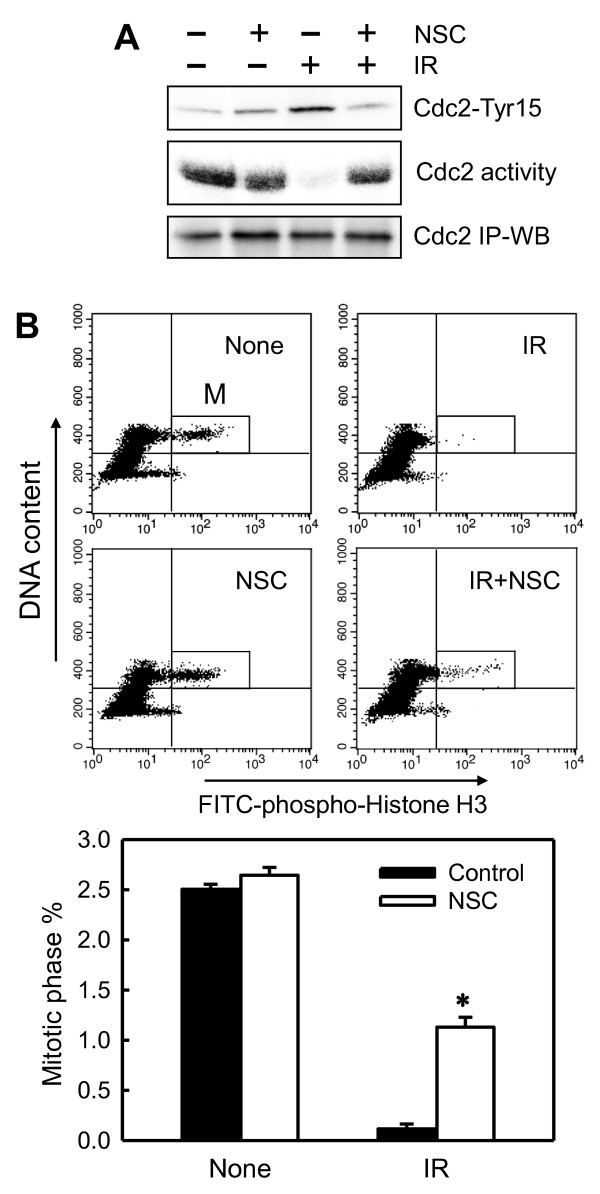
**Rac1 inhibition abrogates IR-induced G_2_/M checkpoint activation**. **(A) **MCF-7 cells were incubated for 1 hour in the presence or absence of 100 μ*M *NSC23766, treated with/without 20-Gy IR, and incubated for an additional 2 hours at 37°C. Cdc2 was immunoprecipitated from cell lysates and analyzed for Cdc2-Tyr15 phosphorylation by immunoblotting (Cdc2-Tyr15) and Cdc2 activity by kinase assay (Cdc2 activity). Amount of Cdc2 protein in the immunoprecipitates was assessed by immunoblotting (Cdc2 IP-WB). **(B) **Cells were treated as described and analyzed for mitotic cells with fluorescence-activated cell sorting (FACS), which contains both 4*N*-DNA content and Histone H3-Ser10 phosphorylation, as described in Materials and methods. Upper panel: the histograms shown are representative FACS analyses for mitotic cells in samples treated with/without IR in the presence or absence of NSC23766. The location of mitotic cells in each sample is indicated (M). Lower panel: the bar graph depicts the percentage of mitotic cells and is shown as mean ± SD of triplicate samples. *P = < 0.001 (*n *= 3), significant difference from cells exposed to IR in the absence of NSC23766.

Because DNA damage-induced G_2_/M checkpoint activation involves phosphorylation of Cdc2-Tyr15 and concomitant inhibition of Cdc2 kinase activity [4-6], we examined the effect of Rac1 inhibition on Cdc2-Tyr15 phosphorylation and Cdc2 activity in IR-treated cells. As shown in Figure 3A, a marked increase was seen in Cdc2-Tyr15 phosphorylation and inhibition of Cdc2 activity within 1 hour after IR exposure of MCF-7 cells. Furthermore, the IR-induced increase in Cdc2-Tyr15 phosphorylation was completely inhibited by the incubation of cells with NSC23766 before IR exposure (Cdc2-Tyr15), and this, in turn, resulted in a complete abrogation of IR-caused inhibition of Cdc2 activity (Figure 3A, Cdc2 activity). Thus, Rac1 activity is apparently necessary for IR-induced Cdc2-Try15 phosphorylation and inhibition of Cdc2 activity.

By using histone-H3 phosphorylation as a marker of cells in mitosis [48], we examined the effect of Rac1 on the proportion of cells in mitosis after IR exposure. As shown in Figure 3B, IR exposure resulted in a rapid decrease in the proportion of cells in mitosis in MCF-7 cells. At 2 hours after IR treatment of MCF-7 cells, an approximate 90% decrease was noted in mitotic cells relative to control nonirradiated cells (Figure 3B, solid bars). In contrast, incubation of cells with NSC23766 blocked the effect of IR, resulting in a significant increase in the proportion of mitotic cells in irradiated cells compared with the control irradiated cells (Figure 3B, IR: open bar versus solid bar). Incubation of cells with NSC23766 alone resulted in a slight increase in the amount of mitotic cells compared with the control untreated cells (Figure 3B, None: open bar versus solid bar).

### Rac1 inhibition abrogates IR-induced ATM and ATR signaling activation

**Figure 4:**
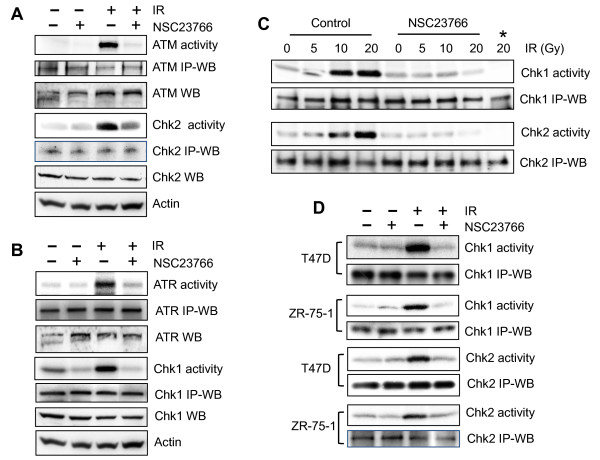
**Rac1 inhibition abolishes IR-induced activation of both ATM and ATR signaling**. **(A) **MCF-7 cells were treated with/without 20-Gy IR in the presence or absence of 100 μ*M *NSC23766 and incubated for 1 hour at 37°C before analysis. To assess the ATM kinase activity, ATM was immunoprecipitated from cell lysate by using anti-ATM antibody (2C1) and assayed for ATM activity by using p53 recombinant protein as substrate. To measure the Chk2 activity, Chk2 was immunoprecipitated from cell lysate by using B-4 anti-Chk2 antibody and assayed for Chk2 activity by using Cdc25C recombinant protein as substrate. As controls, ATM and Chk2 protein levels in the immunoprecipitates (IP-WB) as well as in cell lysates (WB) were assessed with immunoblotting. **(B) **ATR and Chk1 were immunoprecipitated from cell lysates by using N-19 anti-ATR and G-4 anti-Chk1 antibody, respectively. ATR activity was assayed by using p53 recombinant protein substrate, and Chk1 activity assayed by using Cdc25C recombinant protein substrate. As controls, ATR and Chk1 protein levels in the immunoprecipitates (IP-WB), as well as in cell lysates (WB) were assessed with immunoblotting. **(C) **MCF-7 cells were exposed to IR at the indicated doses in the presence or absence of NSC23766, incubated for 1 hour, and assessed for Chk1 and Chk2 activities. *Kinase assay does not contain Cdc25C substrate. **(D) **T47D and ZR-75-1 cells were exposed to 10-Gy IR in the presence or absence of 100 μ*M *NSC23766, incubated for 1 hour, and analyzed for Chk1 and Chk2 activities.

To investigate the mechanisms involved in the regulation of IR-induced G_2_/M checkpoint activation by Rac1, we examined the effect of Rac1 on IR-induced activation of ATM and ATR signaling. For these studies, MCF-7 cells incubated in the presence or absence of NSC23766 were exposed to IR and then examined for the activities of ATM, ATR, Chk1, and Chk2 kinases. As shown in Figure 4A and 4B, incubation of MCF-7 cells with NSC23766 before IR exposure resulted in marked diminution of IR-induced activation of ATM, ATR, Chk1, and Chk2 activities. To verify these effects by Rac1 inhibition, MCF-7 cells were exposed to increasing doses of IR in the presence or absence of NSC23766 and analyzed for Chk1 and Chk2 activities. As shown in Figure 4C, whereas IR exposure of cells resulted in dose-dependent increase in both Chk1 and Chk2 activities, the effect was markedly diminished by the presence of Rac1 inhibition. Furthermore, as shown in Figure 4D, NSC23766 preincubation also abrogated IR-induced Chk1 and Chk2 activation in T47D and ZR-75-1 cells.

### Inhibition of Rac1 by N17Rac1 dominant-negative mutant or Rac1 siRNAs attenuates IR-induced G_2_/M checkpoint activation

**Figure 5:**
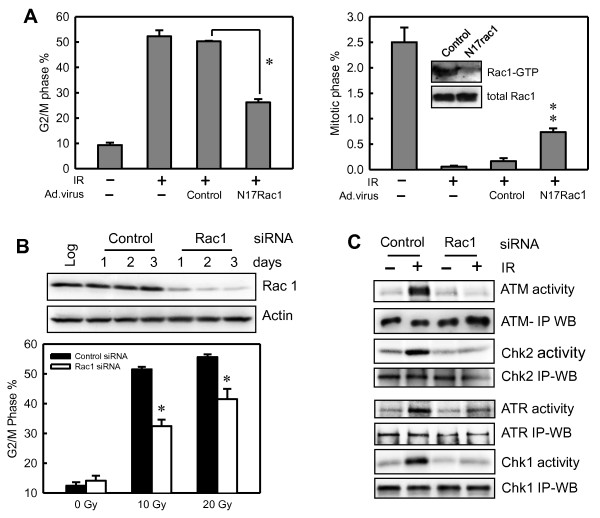
**Inhibition of Rac1 by N17Rac1 mutant or Rac1 siRNA diminishes IR-induced G_2_/M checkpoint activation**. **(A) **MCF-7 cells were infected with Ad.N17Rac1 or Ad.Control for 24 hours and exposed to 15-Gy IR. Left panel: the cells were analyzed for DNA content 24 hours after IR. The result depicts the percentage of cells with *4N*-DNA content and is shown as mean ± SD of quadruplicate samples. *P < 0.001 (*n *= 4), significant difference from the irradiated Ad.Control-infected cells. Right panel: Inset: at 15 minutes after IR, the infected cells were analyzed for Rac1 activities (Rac1-GTP) and protein levels (total Rac1). Bar graph: mitotic cells in the cell samples were analyzed 2 hours after IR. The result depicts the percentage of mitotic cells and is shown as mean ± SD of triplicate samples. **P = 0.002 (*n *= 3), significant difference from the irradiated Ad.Control-infected cells. **(B) **Upper panel: MCF-7 cells transfected with Rac1 siRNA (Rac1) or control siRNA (Control) were incubated for the indicated times and analyzed for protein levels of Rac1 and Actin. Lower panel: After 2-day incubation, the siRNA-transfected cells were exposed to IR, incubated for 24 hours, and assessed for DNA content. Results depict the percentage of cells with *4N*-DNA content and represent the mean ± SD of three separate experiments in duplicate samples. *P < 0.001 (*n *= 6), significant difference from the irradiated Control-siRNA transfected cells. **(C) **After 2-day incubation, siRNA-transfected cells were treated with/without 20-Gy IR, incubated for 1 hour, and analyzed for ATM, ATR, Chk1, and Chk2 activities.

By using an adenoviral vector expressing N17Rac1 dominant-negative mutant [32], we further studied the effect of Rac1 on IR-induced G_2_/M checkpoint response in MCF-7 cells. As shown in Figure 5A (right panel inset), Rac1 assay revealed a much lower Rac1 activity in the irradiated cells expressing N17Rac1 mutant compared with control irradiated cells (N17rac1 versus Control). We next examined the effect of N17Rac1 mutant on IR-induced G_2_/M arrest in MCF-7 cells. As shown in Figure 5A (left bar graph), FACS analyses revealed a marked induction in IR-induced G_2_/M arrest in both noninfected and Ad.Control-infected MCF-7 cells and that this was blocked by the expression of N17Rac1 (N17Rac1 vs. Control, *P *< 0.001; *n *= 4). We also examined the effect of N17Rac1 on the proportion of mitotic cells after IR exposure of MCF-7 cell. As shown in Figure 5A (right panel, bar graph), although a marked decrease in proportion of mitotic cells was found in both noninfected and Ad.Control-infected cells at 2 hours after IR, the expression of N17Rac1 apparently blocked this effect of IR, resulting in a significant increase in amount of mitotic cells compared with Ad.Control-infected cells treated with IR (N17Rac1 vs. Control, *P *= 0.002; *n *= 3).

By using Rac1-specific siRNA, we examined the influence of Rac1 expression on the IR-induced G_2_/M checkpoint response in MCF-7 cells. For these studies, MCF-7 cells were transfected with Rac1-specific siRNA or control nontargeting siRNA and incubated at 37°C for the indicated times. As shown in Figure 5B (upper panel), a 77% reduction in Rac1 protein occurred at 2 days after transfection of cells with Rac1 siRNA. In contrast, transfection of MCF-7 cells with nontargeting control siRNA had no effect on Rac1 protein levels relative to nontransfected cells (Figure 5B, upper panel: Control versus Log). To examine the effect of Rac1 on IR-induced G_2_/M arrest, MCF-7 cells transfected with Rac1 or Control siRNA were exposed to IR at the indicated doses and analyzed for G_2_/M DNA content with FACS. As shown in Figure 5B (lower panel), cells transfected with Rac1 siRNA revealed a marked attenuation in IR-induced G_2_/M arrest compared with control siRNA-transfected cells (open bars versus solid bars; *P *< 0.001; *n *= 6).

We next examined the effect of Rac1 on IR-induced ATM and ATR signaling. As shown in Figure 5C, siRNA-transfected MCF-7 cells exhibited a marked diminution in the activation of ATM, ATR, Chk1, and Chk2 kinases after IR exposure (ATM activity, Chk2 activity, ATR activity and Chk1 activity). In contrast, transfection of MCF-7 cells with control siRNA had no effect on IR-induced activation of ATM, ATR, Chk1 and Chk2 kinases compared with nontransfected control cells (data not shown).

### Rac1 inhibition abolishes IR-induced activation of MEK1/2 and ERK1/2

**Figure 6:**
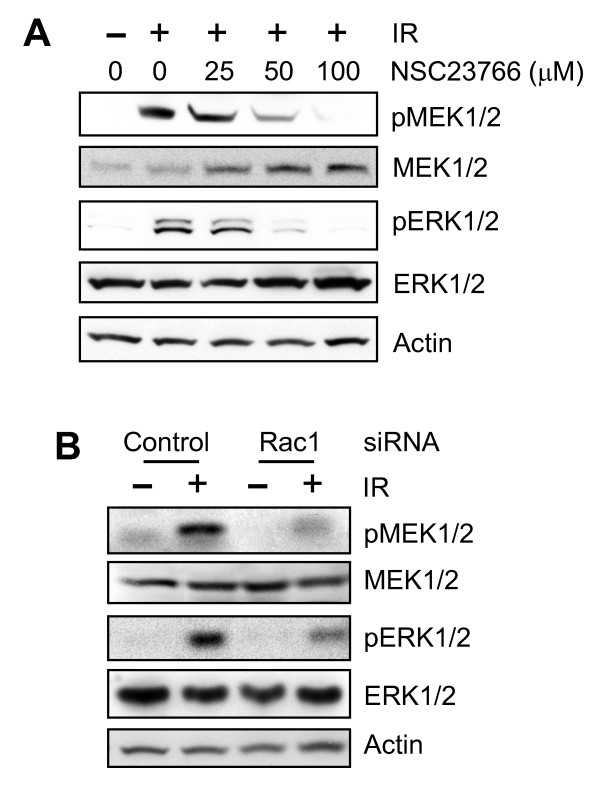
**Rac1 inhibition abolishes IR-induced activation of MEK1/2 and ERK1/2**. **(A) **MCF-7 cells were incubated with increasing doses of NSC23766 for 1 hour at 37°C, exposed to 20-Gy IR, and incubated for additional 15 minutes. The cells were analyzed with immunoblotting for levels of phospho-MEK1/2 (pMEK1/2), phospho-ERK1/2 (pERK1/2), total MEK1/2 (MEK1/2), total ERK1/2 (ERK1/2), and Actin (Actin). **(B) **Cells were transfected with control or Rac1 siRNA, incubated for 2 days, and then exposed to 20-Gy IR or left nonirradiated. After incubation for 15 minutes at 37°C, the cells were examined for levels of phospho-MEK1/2, phospho-ERK1/2, total MEK1/2, total ERK1/2, and actin.

Previous studies from our laboratory demonstrated that IR exposure of cells results in activation of ERK1/2 signaling. Furthermore, IR-induced ERK1/2 signaling is required for G_2_/M checkpoint activation after IR [16]. We therefore examined the effect of Rac1 on IR-induced ERK1/2 signaling activation. For these studies, MCF-7 cells were incubated for 1 hour with increasing doses of NSC23766 and then exposed to 20-Gy IR. At 15 minutes after IR, the cells were examined for MEK1/2 and ERK1/2 phosphorylations by Western blot analysis. As shown in Figure 6A, incubation of cells with Rac1 inhibitor NSC23766 resulted in a dose-dependent diminution of IR-induced phosphorylation of both MEK1/2 and ERK1/2 (pMEK1/2 and pERK1/2). The maximal diminution of IR-induced MEK1/2 and ERK1/2 phosphorylation occurred after incubation of cells with 100 μ*M *NSC23766 (pMEK1/2 and pERK1/2). Furthermore, these changes in phosphorylation of MEK1/2 and ERK1/2 did not involve changes in levels of MEK1/2 and ERK1/2 proteins (Figure 6A, MEK1/2 and ERK1/2).

With Rac1-specific siRNA, the effect of Rac1 expression on IR-induced phosphorylation of MEK1/2 and ERK1/2 was also examined. As shown in Figure 6B, IR-induced phosphorylation of MEK1/2 and ERK1/2 was attenuated in Rac1 siRNA-transfected cells, but not in control siRNA-transfected cells (pMEK1/2 and pERK1/2).

### Inhibition of Rac1 sensitizes MCF-7 cells to IR exposure

**Figure 7:**
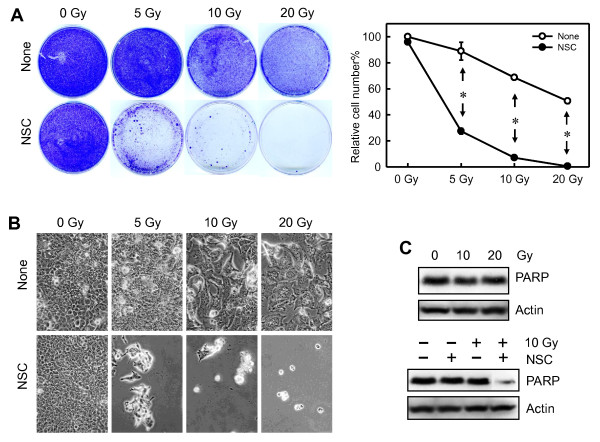
**Inhibition of Rac1 sensitizes MCF-7 cells to irradiation**. **(A) **MCF-7 cells were exposed to increasing doses of IR in the presence or absence of 100 μ*M *NSC23766 and incubated for 3 hours after IR. The cells were washed, incubated in regular growth medium for 7 days, and analyzed for viable cells with crystal violet staining. Left panel: representative sample dishes stained with crystal violet. Right panel: amount of viable cells in each sample was quantified with the ImageJ analytic program, and the results expressed as the mean ± SD of triplicate samples (right panel). *P = < 0.001 (*n *= 3). **(B) **A parallel set of cell samples from this treatment were photographed by using phase-contrast optics. **(C) **Upper panel: cells were exposed to IR at the indicated doses, incubated for 3 days, and analyzed for levels of full-length PARP by immunoblotting (PARP). As a control, Actin levels in cell lysates were assessed with immunoblotting (actin). Lower panel: cells exposed to 10-Gy IR in the presence or absence of NSC23766 were incubated for 3 days and analyzed for levels of full-length PARP (PARP) and actin (actin) with immunoblotting.

As shown in Figures 1 through 5, although IR exposure induced G_2_/M cell-cycle arrest in human breast cancer cells, this effect of IR was markedly attenuated by the Rac1 inhibition. We therefore examined the effect of Rac1 on cell survival after IR exposure. As shown in Figure 7A, IR exposure of MCF-7 cells resulted in dose-dependent decrease in the amount of cells remaining on the culture dish at 7 days after irradiation. Furthermore, IR exposure of cells in the presence of NSC23766 resulted in a further decrease in the amount of cells remaining on the culture dish compared with the samples treated with IR only (Figure 7A). As shown in Figure 7A (right panel), samples exposed to IR in the presence of NSC23766 revealed an additional > 60% decrease in the amount of cells remaining on the culture dish compared with samples exposed to the same dose of IR in the absence of NSC23766. In contrast, samples treated with NSC23766 alone in the absence of IR treatment had no effect on the amount of cells on a culture dish compared with control untreated samples (Figure 7A).

A parallel set of cell samples described earlier was also examined for morphology by using phase-contrast microscopy. As shown in Figure 7B, after 7-day incubation after IR, whereas cells treated with IR alone remained attached to the dish, cells exposed to IR in the presence of NSC23766 rounded up. These results are consistent with those presented in Figure 7A, suggesting that inhibition of Rac1 reduces the survival of MCF-7 cells after IR exposure.

To investigate the possible mechanism involved in the effect of Rac1 inhibition on cell survival after IR exposure, we assessed the integrity of PARP in cells exposed to IR in the presence or absence of NSC23766. Previous studies have shown that the cleavage of PARP, a hallmark of apoptosis, occurs during the execution phase of programmed cell death [49]. As shown in Figure 7C, exposure to increasing doses of IR in the absence of NSC23766 had no detectable effect on the levels of intact PARP in MCF-7 cells, determined at 3 days after IR (Figure 7C, upper panel). In contrast, exposure of cells to IR in the presence of NSC23766 resulted in a marked decrease in levels of intact PARP. These results suggest that the increase in sensitivity of MCF-7 cells to irradiation by NSC23766 involves induction of apoptosis.

## Discussion

G_2_/M transition of the cell cycle is tightly controlled by the activity of the Cdc2/cyclin B complex, which is required for cell entry into mitosis. It has previously been shown that DNA damage induces phosphorylation of Cdc2-Tyr15, resulting in inhibition of Cdc2/cyclin B activity and ultimately G_2_/M arrest [3]. The results in this report indicate that IR exposure of MCF-7 cells induces Rac1 activation (see Figure 1C). Furthermore, inhibition of Rac1 by using the specific inhibitor, dominant-negative mutant Rac1 or specific Rac1 siRNA markedly attenuates IR-induced G_2_/M arrest (see Figures 2 and 5). Additional studies in this report indicate that the inhibition of IR-induced Rac1 activation abolishes IR-induced activation of Chk1 and Chk2 kinases (see Figures 4 and 5) and subsequent Cdc2-Tyr15 phosphorylation (see Figure 3A). Because previous studies indicate that the transition of cells from G_2 _to M phase of the cell cycle requires Cdc2/cyclin B activity, we also assessed the effect of Rac1 inhibition on the proportion of cells in mitosis. The studies presented in Figures 3B and 5A indicate that IR exposure of log-phase growing MCF-7 cells results in a marked decrease in mitotic cells within 2 hours after IR, and that this effect is significantly inhibited by the incubation of cells with NSC23766 or expression of the N17Rac1 dominant-negative mutant. Thus, Rac1 inhibition diminishes IR-induced G_2_/M checkpoint activation and increases the entry of cells from G_2 _into M phase of the cell cycle in MCF-7 cells exposed to IR. These studies suggest Rac1 as an upstream regulator of G_2_/M checkpoint response after exposure of cells to IR.

Cellular response to IR-induced DNA damage involves activation of ATM and ATR signaling, which results in activation of the Wee1 kinase that phosphorylates Cdc2-Tyr15 and inhibition of the Cdc25 phosphatase that dephosphorylates Cdc2-Tyr15 [50,51]. Although it still remains unclear how exactly the ATM and ATR kinases are activated in response to genotoxic stress, evidence suggests that multiple mechanisms might be involved in the regulation of this biologic process. Supporting this speculation, a recent study by Wang *et al. *[52] reported that the p38MAPK pathway is required for the activation of ATR kinase after expression of hepatitis B virus X protein.

Another example is NBS1, a component of the MRE11/RAD50/NBS1 complex, which not only is involved in certain downstream steps of ATM- and ATR-dependent DNA damage response but also functions as an upstream mediator required for the ATM and ATR signaling activation after IR-induced DNA damage [53]. The results from the present report suggest that Rac1 also plays an important role in the activation of ATM and ATR signaling after IR exposure of cells (see Figures 4 and 5).

A previous study demonstrated that incubation of MCF-7 cells with Rac1 specific inhibitor NSC23766 at 100 μ*M *for 48 hours results in a G_1 _cell-cycle arrest [54]. However, in the present studies, we observe that incubation of MCF-7 cells with 100 μ*M *NSC23766 for up to 24 hours does not result in a detectable increase in G_1_-phase cells relative to control untreated cells (see Additional file 1, Figure S1). Furthermore, incubation of other cells, including MDA-MB-231, T47D, and ZR-75-1, with 100 μ*M *NSC23766 for up to 24 hours, also does not result in an increase in percentage of G_1_-phase cells (data not shown). Thus, the effect of NSC23766 on G_1_-phase cells is probably time dependent. Additional studies are needed to understand the effect of prolonged Rac1 inhibition on cell-cycle regulation in log-phase growing cells.

Expression of N17Rac1 dominant-negative mutant for 72 hours has been previously shown to result in G_2_/M cell-cycle arrest in Rat 2 fibroblast cells [32]. In the present studies, after 24-hour expression of N17Rac1, we do not observe any noticeable effect by N17Rac1 on the proportion of G_2_/M phase cells in log-phase growing MCF-7 cells (data not shown). Thus, the effect of N17Rac1 on G_2_/M phase cells is probably cell-type specific and/or time dependent. In contrast, expression of N17Rac1 in MCF-7 cells abrogates the IR-induced activation of Rac1, and this, in turn, is associated with an attenuation of G_2_/M arrest in irradiated cells and an increase in the amount of mitotic cells after irradiation (see Figure 5A).

Previous studies from several laboratories, including our own, have suggested an important role for ERK1/2 signaling in the activation of the G_2_/M checkpoint response after DNA damage [16,20,21]. These studies have demonstrated that DNA damage induces ERK1/2 activation and that this is associated with the induction of G_2_/M arrest. Additional studies demonstrate that inhibition of ERK1/2 abrogates the G_2_/M checkpoint response after DNA damage, resulting in increased sensitivity of cells to DNA-damaging agents [16,20,21]. Results presented in this report indicate that Rac1 inhibition after incubation of cells with a specific inhibitor or transfection with Rac1-specific siRNA abrogates IR-induced phosphorylation of MEK1/2 and ERK1/2 (see Figure 6), as well as the IR-induced G_2_/M checkpoint activation (see Figures 2 through 5), suggesting Rac1 as the upstream regulator of IR-induced ERK1/2 signaling.

A role for p53 in the regulation of the G_2_/M checkpoint response has been suggested by previous studies, as several of the transcriptional targets of p53 can directly or indirectly inhibit Cdc2 kinase, which include p21^Waf1/Cip1^, 14-3-3σ, and Gadd45 [55]. However, the results of this report suggest that IR-induced G_2_/M cell-cycle arrest as well as the regulation of Rac1 on the IR-induced G_2_/M checkpoint response is apparently independent of p53, as among the four breast cancer cell lines used for the studies, MDA-MB-231 and T47D cells express mutant p53 [56], whereas MCF-7 and ZR75-1 express wild-type p53. Consistent with our observation, results from other studies also show that p53 status has no influence on IR-induced G_2_/M cycle arrest [57].

The results in Figures 2 through 5 show that IR-induced G_2_/M arrest in human breast cancer cells is markedly attenuated by the inhibition of Rac1. Furthermore, the results in Figure 7 and Additional file 1, Figure S4, provide evidence that Rac1 inhibition significantly increases the sensitivity of MCF-7 cells to irradiation, which involves apoptosis induction. These results suggest a strong correlation between the attenuation of G_2_/M arrest and the increased radiation sensitivity in MCF-7 cells treated with IR in the presence of Rac1 inhibition. It is possible that the increased radiation sensitivity is simply a consequence of the attenuation of IR-induced G_2_/M arrest by Rac1 inhibition. However, it could also be due to a new function of Rac1. Future studies must address this question.

In this report, we also tested the effect of Rac1 inhibition on IR-induced G_2_/M arrest in normal human mammary epithelial cells (MCF-10A and 76N). The results are unexpected, as the Rac1 inhibition by NSC23766 does not block the IR-induced G_2_/M arrest in these cells (see Additional file 1, Figure S5), whereas it blocks completely the IR-induced G_2_/M arrest in human breast cancer cells (see Figures 2 through 5). The mechanism causing this difference is unclear. However, it should be noted that the growth medium DFCI-1 used for the normal human mammary epithelial cells contains additional growth factors that are not presented in the medium for maintaining the breast cancer cells, which include EGF, estradiol, and insulin (see Materials and methods). It might be that these additional components in DFCI-1 growth medium compensate for the effect of Rac1 inhibition on IR-induced G_2_/M checkpoint activation. We will investigate this possibility in future studies.

Previous studies from our laboratory demonstrate that inhibition of ERK1/2 by MEK1/2 specific inhibitors or decreased ERK1/2 expression by transfection of cells with ERK1/2 siRNA abrogated the IR-induced ATR activation in MCF-7 cells but had little effect on ATM activation [16]. Furthermore, additional studies demonstrate that ERK1/2 signaling is upstream of ATR, as decreased ATR expression in MCF-7 cells after transfection with ATR siRNA or incubation of cells with caffeine, which inhibits both ATR and ATM, has no effect on IR-induced ERK1/2 activation [16]. Results presented in this study indicate that Rac1 activation not only is necessary for the activation of ERK1/2 and ATR kinases, but also is essential for the activation of ATM signaling after IR exposure (Figures 4 and 5).

A growing amount of evidence shows that IR exposure of breast cancer cells frequently results in G_2_/M cell-cycle arrest [58], and induction of cell-cycle arrest after DNA damage has been associated with DNA repair and cell survival [50,59,60]. Thus, a better understanding of the mechanisms responsible for IR-induced G_2_/M cell-cycle arrest would potentially allow identifying novel therapeutic targets that could be exploited to sensitize breast cancer cells to radiation treatment.

Results in this report provide evidence supporting a novel role for Rac1 in the activation of G_2_/M checkpoint response and promotion of cell survival after IR exposure.

## Conclusions

IR exposure of MCF-7 breast cancer cells was associated with a rapid activation of Rac1 and an induction of G_2_/M cell cycle arrest. Furthermore, inhibition of Rac1 by using specific inhibitor, dominant-negative mutant Rac1 or specific siRNA diminished IR-induced activation of ATM and ATR signaling and attenuated IR-induced G_2_/M cell cycle arrest. Moreover, inhibition of Rac1 markedly increased the sensitivity of MCF-7 cells to IR exposure, which involves induction of apoptosis. Collectively, results in this report suggest an important role of Rac1 in the activation of the G_2_/M checkpoint response and cell survival after IR exposure.

## Abbreviations

ATM: ataxia telangiectasia mutated; ATR: ATM- and rad3-related; DMSO: dimethyl sulfoxide; ERK: extracellular signal-regulated protein kinase; pERK: phosphorylated-extracellular signal-regulated protein kinase; FACS: fluorescence-activated cell sorting; GTPases: guanosine triphosphatases; IR: γ-irradiation; MEK1/2: mitogen-activated protein kinase kinase 1 and 2; PI: propidium iodide; Rac1: Ras-related C3 botulinum toxin substrate 1.

## Competing interests

The authors declare that they have no competing interests.

## Authors' contributions

Experiments were performed by PG, PC, RK, and YY. The studies were designed, analyzed, and interpreted by YY and KC. The manuscript was drafted by YY and critically revised by KC. All authors read and approved the final manuscript.

## Supplementary Material

Additional file 1 **Figure S1**. Effect of NSC23766 on cell cycle after IR exposure of MCF-7 cells. **Figure S2**. Incubation with NSC23766 did not result in apoptosis induction in MCF-7 cells. **Figure S3**. Treatment with NSC23766 had no effect on clonogenic survival of MCF-7 cells. **Figure S4**. Inhibition of Rac1 by NSC23766 decreased the ability of irradiated MCF-7 cells to grow colonies. **Figure S5**. Inhibition of Rac1 by NSC23766 had no effect on IR-induced G_2_/M arrest in normal human mammary epithelial cells. Click here for file
