# Supplementary material for: RAC1 GTPase plays an important role in γ-irradiation induced G2/M checkpoint activation
Source: Breast Cancer Res. 2012 Apr 11;14(2):R60. doi: 10.1186/bcr3164 (PMC3446395; doi:10.1186/bcr3164)
Supplement: Additional file 1 — Figure S1. Effect of NSC23766 on cell cycle after IR exposure of MCF-7 cells. Figure S2. Incubation with NSC23766 did not result in apoptosis induction in MCF-7 cells. Figure S3. Treatment with NSC23766 had no effect on clonogenic survival of MCF-7 cells. Figure S4. Inhibition of Rac1 by NSC23766 decreased the ability of irradiated MCF-7 cells to grow colonies. Figure S5. Inhibition of Rac1 by NSC23766 had no effect on IR-induced G2/M arrest in normal human mammary epithelial cells. [file bcr3164-S1.PDF]

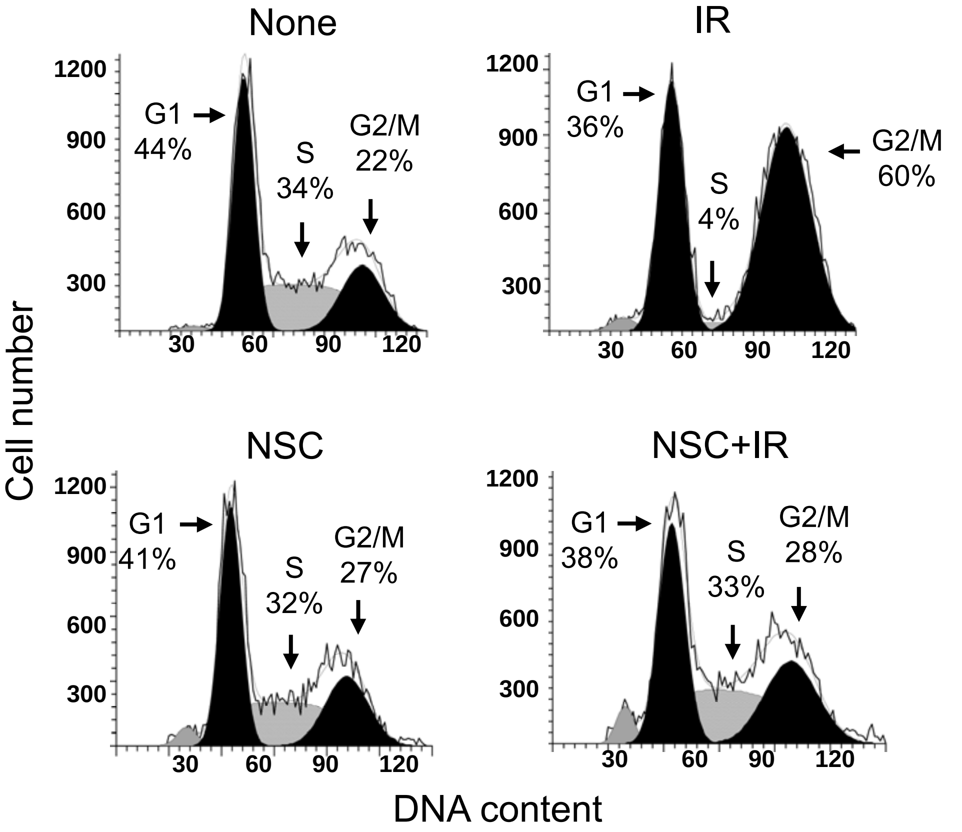

Figure S1. Effect of NSC23766 on cell cycle following IR exposure of MCF-7 cells. MCF-7 cells were pre-incubated for 1 hr in the presence or absence of 100 μM NSC23766 and then exposed to 20-Gy IR or, as a control, left non-irradiated. Following incubation at 37°C for 24 hr post IR, the cells were analyzed for DNA content by FACS. The histograms shown are representative FACS analyses of quadruplicate cell samples. Amounts of cells in G1, S and G2/M phase of the cell cycle are indicated.

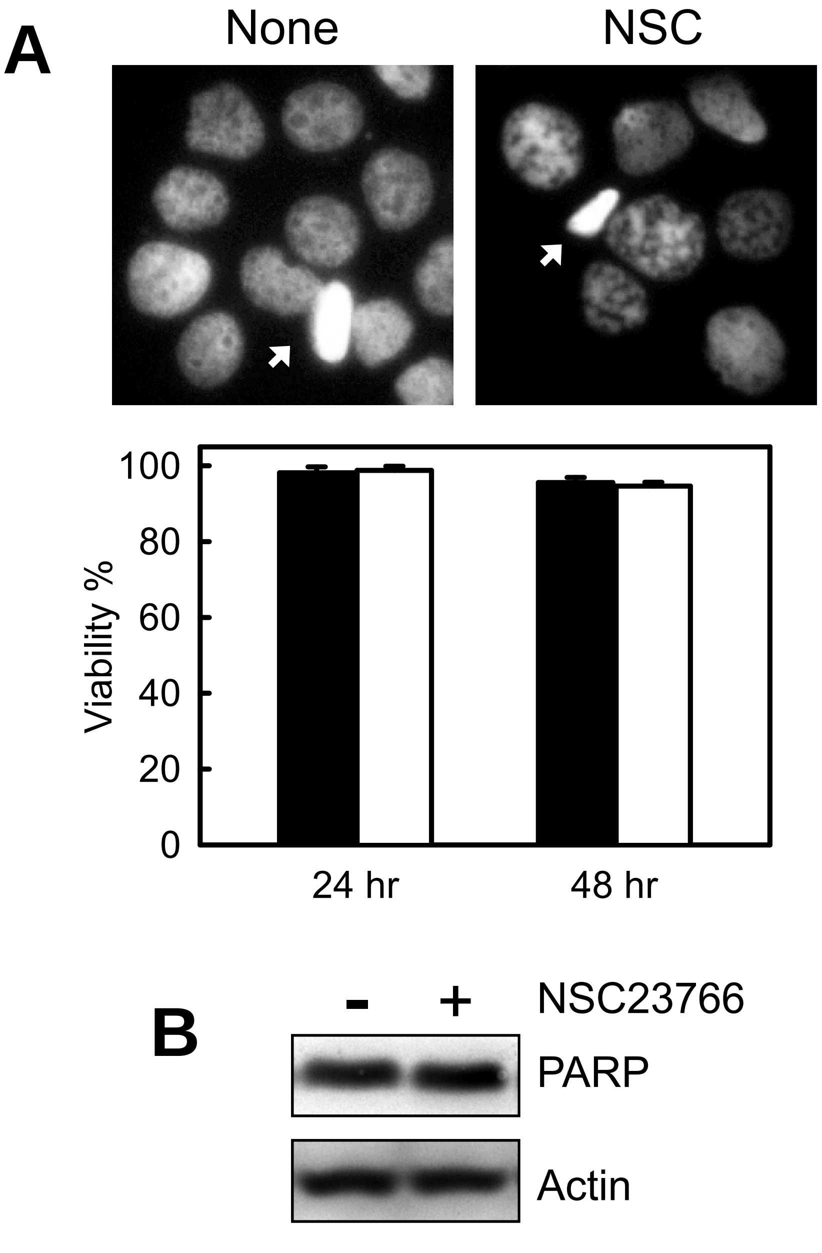

Figure S2. Incubation with NSC23766 does not result in apoptosis induction MCF-7 cells. A. MCF-7 cells were incubated with 100 μM NSC23766 for 24 hr or 48 hr and analyzed for cell survival by DAPI staining and fluorescence microcopy as described in *Materials and methods*. Upper panel: representative DAPI staining analyses of the MCF-7 cells incubated for 24 hr in the absence (*None*) or presence of NSC23766 (*NSC*). Lower panel: percentage of viable cells is shown as mean±s.d of quadruplicate samples. Solid bars: control non-treated cells; open bars: NSC23766 treated cells. B. Levels of full-length PARP in the resulting cells (48 hr time point) were analyzed by immunoblotting. Protein loadings were assessed by immunoblotting for Actin levels (*Actin*).

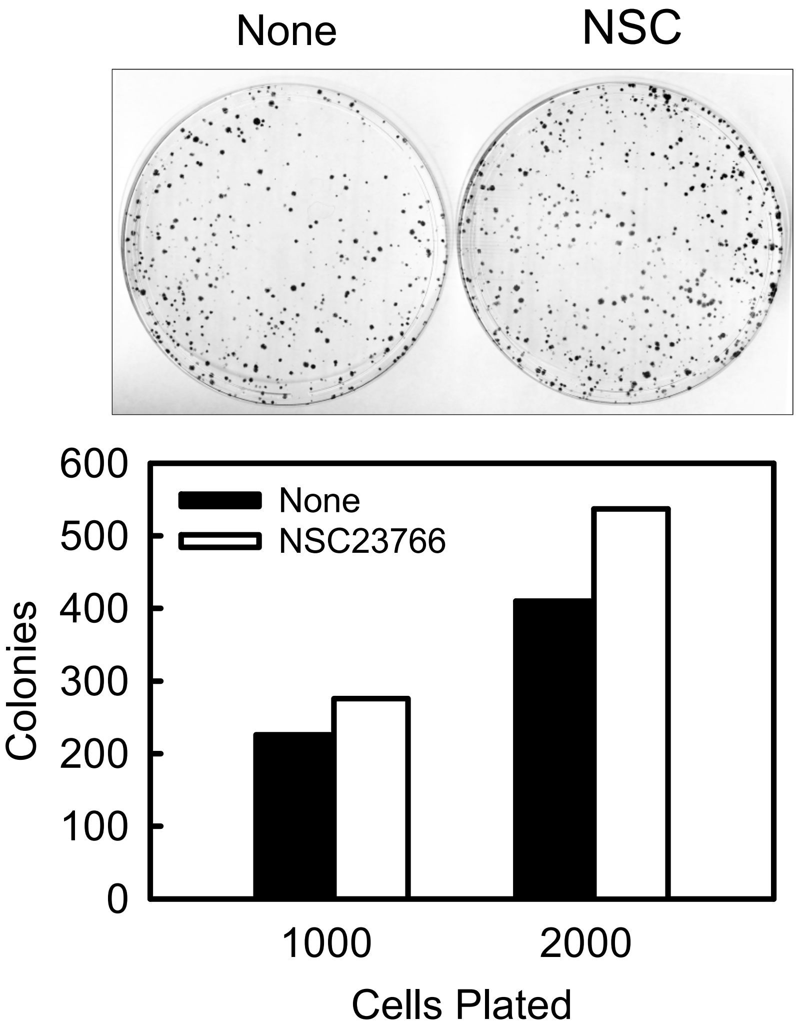

Figure S3. Treatment with NSC23766 has no effect on clonogenic survival of MCF-7 cells. MCF-7 cells treated with or without 100 μM NSC23766 were examined for their ability to grow colonies by clonogenic assay, as described in *Materials and methods*. Upper panels: representative dishes resulted by the clonogenic assay (1000 cells per dish plated). Lower panel: the amounts of colonies in the resulting dishes were quantified using ImageJ analytical program and are shown as average of duplicate samples. The experiment was repeated two times and similar results were attained.

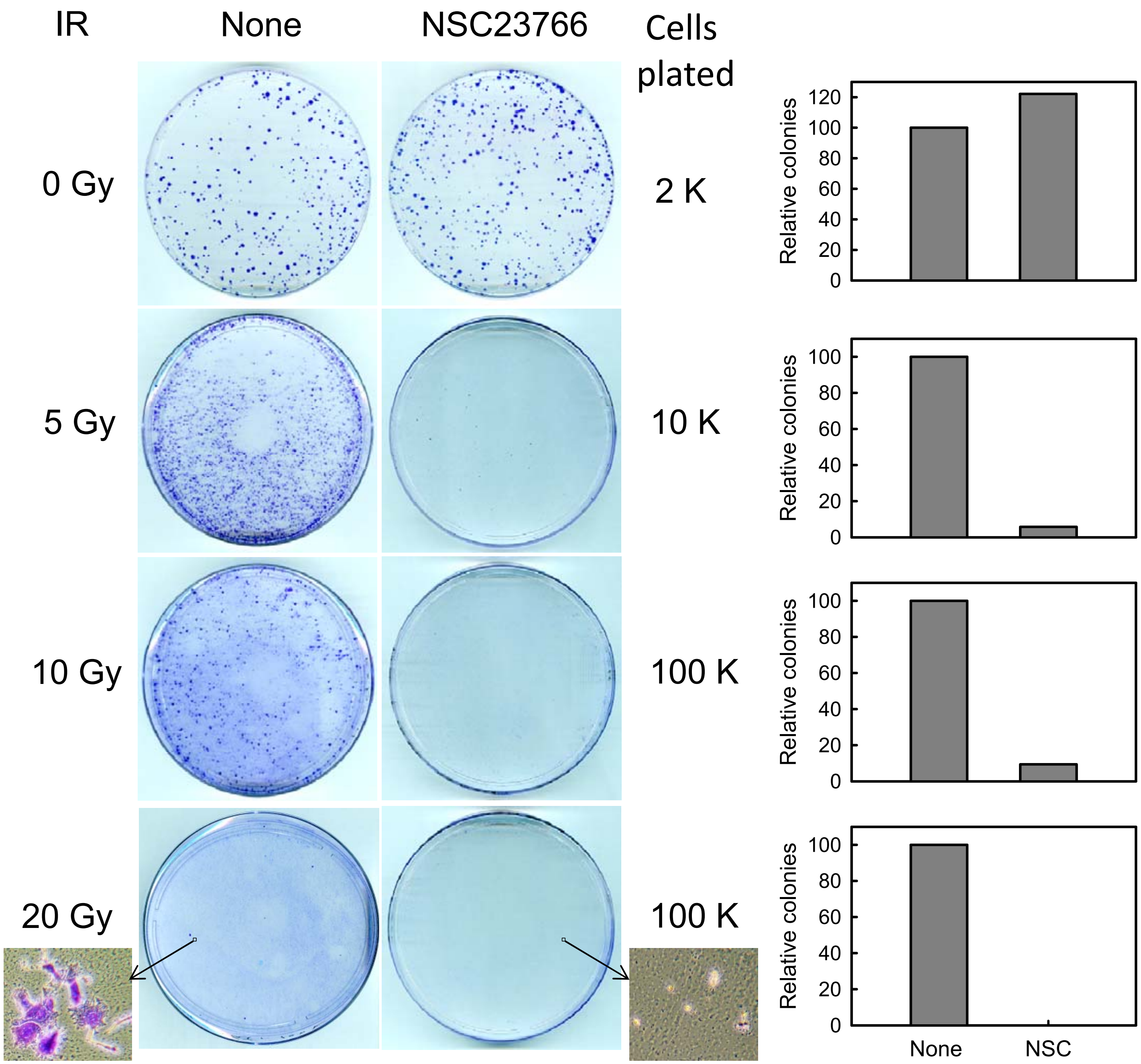

Figure S4. Inhibition of Rac1 by NSC23766 decreases the ability of irradiated MCF-7 cells to grow colonies. MCF-7 cells incubated in the presence or absence of 100 μM NSC23766 were exposed to increasing doses of IR and subsequently examined for their ability to produce colonies by clonogenic assay. Left panels: representative sample dishes from the clonogenic assay stained with crystal violet. Inserts: phase-contrast microscopy cell images (left image, cells exposed to 20-Gy IR; right image, cells exposed to 20-Gy IR in the presence of NSC23766). Right panel: number of colonies in the resulting samples was quantified using ImageJ analytical program and the relative number of colonies in the samples treated with IR only versus the samples treated with both IR and NSC23766 were compared. Results are shown as the average of duplicate samples. The experiment was repeated twice and similar results were attained.

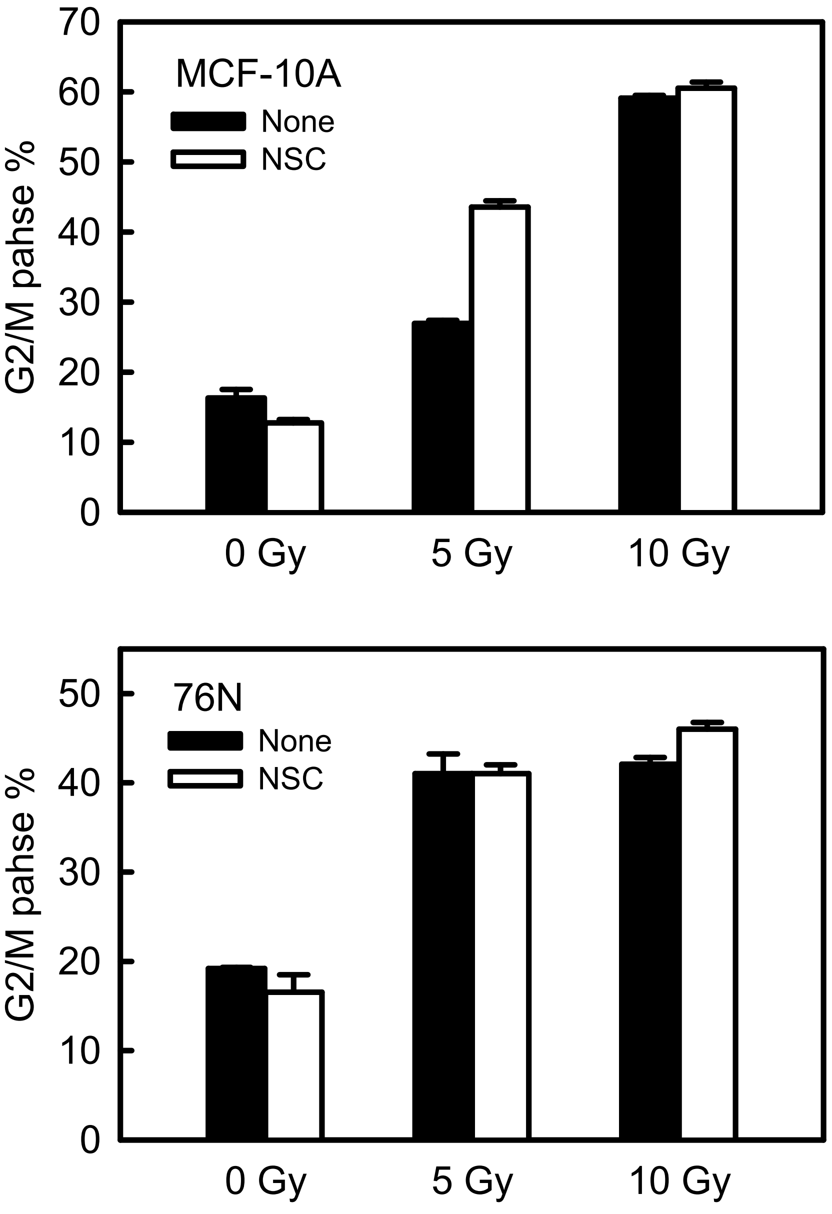

Figure S5. Inhibition of Rac1 by NSC23766 had no effect on IR induced G2/M arrest in normal human mammary epithelia cells. Upper panel: MCF-10A cells were incubated in the presence or absence of 100 μM NSC23766 for 1 hr and exposed to IR at the doses indicated. The cells were incubated for 24 hr and analyzed for DNA content by flow cytometry. The result depicts the percentage of cells with *4N*-DNA content and is shown as mean±s.d. of duplicate cell samples from two separate experiments. Lower panel: 76N cells were treated as described above and assessed for DNA content. The result depicts the percentage of cells with *4N*-DNA content and is shown as mean±s.d. of duplicate cell samples from two separate experiments.
